# Diphtheria antitoxin levels in the Netherlands: a population-based study.

**DOI:** 10.3201/eid0505.990511

**Published:** 1999

**Authors:** H E de Melker, G A Berbers, N J Nagelkerke, M A Conyn-van Spaendonck

**Affiliations:** National Institute of Public Health and the Environment, Bilthoven, The Netherlands. H.de.Melker@rivm.nl

## Abstract

In a population-based study in the Netherlands, diphtheria antitoxin antibodies were measured with a toxin-binding inhibition assay in 9, 134 sera from the general population and religious communities refusing vaccination. The Dutch immunization program appears to induce long-term protection against diphtheria. However, a substantial number of adults born before the program was introduced had no protective diphtheria antibody levels. Although herd immunity seems adequate, long-term population protection cannot be assured. As more than 60% of orthodox reformed persons have antibody levels lower than 0.01 IU/ml, introduction of diphtheria into religious communities refusing vaccination may constitute a danger of spread of the bacterium.


### Diphtheria Antitoxin Levels in the Netherlands: a Population-Based Study

**H.E. de Melker, G.A.M. Berbers, N.J.D. Nagelkerke, and M.A.E. Conyn-van Spaendonck**
National Institute of Public Health and the Environment, Bilthoven, the Netherlands

In a population-based study in the Netherlands, diphtheria antitoxin antibodies were measured with a toxin-binding inhibition assay in 9,134 sera from the general population and religious communities refusing vaccination. The Dutch immunization program appears to induce long-term protection against diphtheria. However, a substantial number of adults born before the program was introduced had no protective diphtheria antibody levels. Although herd immunity seems adequate, long-term population protection cannot be assured. As more than 60% of orthodox reformed persons have antibody levels lower than 0.01 IU/ml, introduction of diphtheria into religious communities refusing vaccination may constitute a danger of spread of the bacterium.

The recent diphtheria epidemics in eastern Europe are a warning that diphtheria can make a comeback in susceptible populations (1). The World Health Organization (WHO) recommends the assessment of diphtheria immunity in nonepidemic countries, to prevent any indigenous cases in the European region by the year 2000 (2).

In the Netherlands, the last diphtheria epidemic occurred during World War II (220,000 cases in 1940 to 1946). Diphtheria vaccination was introduced in 1952 for persons born after 1945. Under the current schedule, children are vaccinated at ages 3, 4, 5, and 11 months with diphtheria, tetanus, pertussis, and inactivated polio vaccine (DTP-IPV) and at ages 4 and 9 years with DT-IPV. For the past 25 years, the vaccine coverage for at least three vaccinations at the age of 12 months has been 97%. Rare exposure to *Corynebacterium diphtheriae* may have led to lack of boosting opportunities (1). As in other industrialized countries, lack of immunity in older persons is a reason for concern (3,4). Furthermore, in the Netherlands, the immune status of sociogeographically clustered members of religious communities who refuse vaccination may be even more unfavorable. Inadequate herd immunity to diphtheria in these groups could lead to outbreaks similar to the poliomyelitis outbreaks in the Netherlands (5). A large population-based serum bank allowed us to assess the diphtheria immunity in the general Dutch population and in persons refusing vaccination (6).

Address for correspondence: H.E. de Melker, National Institute of Public Health and the Environment, Department of Infectious Diseases Epidemiology, P.O. Box 1, 3720 BA Bilthoven, The Netherlands; fax: 31-30-274-4409; e-mail: H.de.Melker@rivm.nl.

### The Study

From October 1995 through December 1996, a population-based serum bank with specimens from 9,948 persons was established (6). Our objective was to select 40 municipalities with samples proportional to population size. In each of five regions, eight municipalities were included. For each of these 40 municipalities, an age-stratified sample of 380 persons was drawn from the population register (7). Participants were requested to have a blood sample drawn, complete a questionnaire, and provide immunization and military service records. Participants were also selected from eight additional municipalities with low vaccine coverage to assess the immunity of members of religious communities that refuse vaccination. The nationwide sample had 8,357 (55%) participants, and the low vaccine coverage sample had 1,589 (52.5%). Sufficient serum was available for testing 7,715 of the nationwide participants and 1,419 of the participants in the sample with low vaccine coverage.

### Methods

Sera were stored at -86°C. The level of diphtheria antitoxin antibodies was measured with a toxin-binding inhibition assay (8). In brief, twofold serum dilution series were incubated with a fixed amount of toxin, and the nonneutralized toxin was measured in an enzyme-linked immunosorbent assay (ELISA) with equine antitoxin purified from hyperimmune serum as coat and peroxidase-labeled horse antidiphtheria IgG as conjugate. International units were calculated according to the WHO reference standard serum (10 IU/ml) by the four-parameter fit method in Kineticalc (KC4, Biolyse) with a Bio-Tek plate reader (EL312d). The minimum level of detection was 0.01 IU/ml, and samples below this level were set to 0.005 IU/ml for calculating geometric mean titers. The correlation of this method with the Vero neutralization assay has been confirmed recently (r ≥ 0.95) (9).

Antitoxin antibody levels were classified according to international standards as < 0.01 IU/ml (no protection), 0.01 IU/ml to 0.1 IU/ml (basic protection) and > 0.1 IU/ml (full protection) (10).

### Analysis

Frequencies and geometric mean titers in each municipality were weighted by the proportion of the age group in the population. To produce national estimates, the weighted frequencies and geometric mean titers were averaged over the 40 municipalities (7). For the low vaccine coverage sample, they were averaged by weighting the population of the municipality.

Data on age, sex, marital status, country of nationality, degree of urbanization, region, and contact information for all participants and nonparticipants were available. The effect of differential probabilities of response for these variables on both sample estimates was less than one standard error and was therefore disregarded.

Linear regression analysis was used to study the persistence of diphtheria antitoxin antibodies after full immunization in the national immunization program. The association between diphtheria antibody titer ($^2$log) and age in $^2$log years was studied for persons who received the sixth documented vaccination at 8 to 9 years of age, without self-reported or documented revaccination or history of military service.

### Age-Specific Immunity Levels to Diphtheria Antitoxin

In the nationwide sample, 58.1%, 30.0%, and 11.9% of persons ≤ 79 years of age had full, basic, or no diphtheria protection, respectively (Table 1). Women had lower levels of full protection and geometric mean titers. A greater percentage of persons from the municipalities with low vaccine coverage and of members of religious communities in the low vaccine coverage sample had no protection (Table 2). When members of the religious community were excluded from this low

Table 1. Diphtheria immunity in a nationwide sample of persons ≤ 79 years of age, the Netherlands

| Sample | No. | <0.01 IU/ml | (95% CI[a]) | 0.01-0.1 IU/ml | (95% CI) | ≥ 0.1 IU/ml | (95% CI) | Geometric mean titer | (95% CI) |
|---|---|---|---|---|---|---|---|---|---|
| Overall | 7,715 | 11.9 | (10.7-13.1) | 30.0 | (28.3-31.7) | 58.1 | (56.2-59.9) | 0.12 | (0.11-0.13) |
| Men | 3,644 | 9.3 | (8.0-10.6) | 28.1 | (25.9-30.2) | 62.6 | (60.1-65.2) | 0.14 | (0.13-0.16) |
| Women | 4,071 | 14.4 | (12.6-16.2) | 31.6 | (29.7-33.6) | 54.0 | (51.9-56.0) | 0.10 | (0.09-0.11) |

[a]CI, confidence interval.

Table 2. Diphtheria immunity in a sample of persons ≤ 79 years of age with low vaccine coverage, the Netherlands

| Nationwide sample[a] | No. | <0.01 IU/ml | (95% CI[b]) | <0.01 IU/ml | (95% CI) | ≥ 0.1 IU/ml | (95% CI) | Geometric mean titer | (95% CI) |
|---|---|---|---|---|---|---|---|---|---|
| **OR** | | | | | | | | | |
| Overall | 233 | 60.9 | (42.9-78.9) | 12.4 | (6.0-18.7) | 26.7 | (13.8-39.7) | 0.02 | (0.01-0.04) |
| Men | 116 | 69.7 | (54.2-85.2) | 10.4 | (4.2-16.6) | 19.9 | (8.2-31.5) | 0.01 | (0.01-0.03) |
| Women | 117 | 52.3 | (27.7-76.8) | 15.0 | (6.9-23.2) | 32.7 | (15.3-50.1) | 0.03 | (0.01-0.06) |
| **NMCR** | | | | | | | | | |
| Overall | 1,259 | 17.5 | (15.5-19.4) | 25.2 | (21.3-29.2) | 57.3 | (52.2-62.4) | 0.10 | (0.09-0.12) |
| Men | 590 | 11.5 | (8.2-14.8) | 25.5 | (20.5-30.6) | 63.0 | (55.4-70.5) | 0.14 | (0.13-0.16) |
| Women | 669 | 21.7 | (18.0-25.4) | 25.0 | (21.2-28.9) | 53.3 | (48.6-58.0) | 0.08 | (0.07-0.10) |
| **TLVC** | | | | | | | | | |
| Overall | 1,492 | 24.3 | (20.5-28.0) | 23.6 | (20.1-27.1) | 52.1 | (48.2-56.0) | 0.08 | (0.07-0.09) |
| Men | 706 | 20.6 | (16.6-24.7) | 23.4 | (18.9-27.8) | 56.0 | (50.3-61.7) | 0.10 | (0.08-0.12) |
| Women | 786 | 26.7 | (21.1-32.4) | 23.3 | (19.9-26.7) | 50.0 | (45.3-54.7) | 0.07 | (0.06-0.09) |

[a]OR, orthodox reformed; NMCR, Not member of religious communities; TLVC, total low vaccine coverage.
[b]CI, confidence interval.

vaccine coverage sample, the percentages of full, basic, and no protection were 57.3%, 25.2%, and 17.5%, respectively (Table 2).

For the ages of 1, 4, and 8 to 9 years, the geometric mean titer and percentages of persons with full protection increased (Table 3). The

Table 3. Age-specific prevalence of diphtheria immunity and geometric mean titers for children ≤ 14 years of age and for men and women ≤ 79 years of age in a nationwide sample, the Netherlands

| Age group (yrs) | No. | <0.01 IU/ml | (95% CI[a]) | 0.01-0.1 IU/ml | (95% CI) | ≥ 0.1 IU/ml | (95% CI) | Geometric mean titer | (95% CI) |
|---|---|---|---|---|---|---|---|---|---|
| <1 | 187 | 3.7 | (1.1-6.4) | 25.5 | (18.6-32.4) | 70.7 | (63.4-78.1) | 0.19 | (0.15-0.24) |
| 1 | 185 | 1.2 | (0.0-3.0) | 9.9 | (5.4-14.3) | 88.9 | (84.3-93.5) | 0.57 | (0.45-0.72) |
| 2 | 156 | 1.9 | (0.1-3.7) | 34.9 | (24.2-45.5) | 63.3 | (52.9-73.6) | 0.15 | (0.12-0.19) |
| 3 | 215 | 1.7 | (0.1-3.3) | 35.5 | (27.7-43.3) | 62.8 | (54.7-70.9) | 0.16 | (0.12-0.22) |
| 4 | 153 | 0.6 | (0.0-1.7) | 8.7 | (3.7-13.7) | 90.7 | (85.7-95.7) | 0.81 | (0.60-1.10) |
| 5 | 102 | 1.4 | (0.0-3.3) | 6.3 | (1.9-10.7) | 92.3 | (87.7-97.0) | 0.43 | (0.35-0.52) |
| 6 | 121 | 0.9 | (0.0-2.6) | 20.0 | (11.7-28.3) | 79.1 | (70.9-87.4) | 0.26 | (0.21-0.34) |
| 7 | 101 | 2.3 | (0.0-5.4) | 31.8 | (20.3-43.3) | 65.9 | (54.7-77.2) | 0.16 | (0.13-0.20) |
| 8 | 127 | 0.7 | (0.0-2.0) | 23.6 | (14.9-32.3) | 75.8 | (66.9-84.6) | 0.28 | (0.20-0.38) |
| 9 | 97 | 0.0 | (0.0-0.0) | 11.8 | (3.2-20.4) | 88.2 | (79.6-96.8) | 0.71 | (0.51-0.99) |
| 10 | 113 | 0.7 | (0.0-2.1) | 6.7 | (1.9-11.5) | 92.6 | (87.7-97.5) | 0.54 | (0.42-0.69) |
| 11 | 111 | 0.7 | (0.0-1.9) | 8.3 | (3.5-13.2) | 91.0 | (85.8-96.2) | 0.36 | (0.30-0.44) |
| 12 | 122 | 0.6 | (0.0-1.9) | 17.0 | (9.2-24.8) | 82.4 | (74.2-90.5) | 0.28 | (0.22-0.35) |
| 13 | 126 | 0.0 | (0.0-0.0) | 27.3 | (18.0-36.7) | 72.7 | (63.3-82.0) | 0.23 | (0.18-0.29) |
| 14 | 102 | 0.4 | (0.0-1.1) | 26.7 | (14.0-39.3) | 72.9 | (60.3-85.6) | 0.22 | (0.15-0.31) |
| **Men** | | | | | | | | | |
| <1 | 104 | 3.5 | (0.04-7.0) | 25.2 | (15.8-34.6) | 71.3 | (61.4-81.2) | 0.18 | (0.13-0.24) |
| 1-4 | 376 | 1.8 | (0.5-3.1) | 22.9 | (17.0-28.8) | 75.3 | (69.1-81.5) | 0.31 | (0.25-0.38) |
| 5-9 | 296 | 0.6 | (0.0-1.4) | 16.9 | (12.4-21.4) | 82.5 | (77.9-87.1) | 0.34 | (0.28-0.40) |
| 10-14 | 280 | 0.6 | (0.0-1.7) | 16.5 | (11.7-21.3) | 83.0 | (78.0-88.0) | 0.32 | (0.27-0.38) |
| 15-19 | 209 | 1.0 | (0.0-2.4) | 25.8 | (19.7-31.9) | 73.1 | (67.1-79.1) | 0.19 | (0.16-0.23) |
| 20-24 | 139 | 4.0 | (0.4-7.6) | 25.5 | (17.8-33.2) | 70.5 | (62.0-79.0) | 0.15 | (0.12-0.19) |
| 25-29 | 150 | 2.9 | (0.0-6.2) | 30.6 | (21.7-39.5) | 66.5 | (56.9-76.1) | 0.18 | (0.14-0.24) |
| 30-34 | 188 | 7.0 | (1.6-12.4) | 31.5 | (23.0-40.0) | 61.5 | (52.1-70.9) | 0.14 | (0.10-0.19) |
| 35-39 | 220 | 5.1 | (1.3-8.9) | 27.4 | (20.7-34.1) | 67.5 | (60.8-74.2) | 0.18 | (0.14-0.23) |
| 40-44 | 230 | 3.8 | (1.1-6.5) | 16.3 | (11.0-21.6) | 79.9 | (73.9-85.9) | 0.25 | (0.20-0.31) |
| 45-49 | 208 | 13.7 | (7.8-19.6) | 26.6 | (21.4-31.8) | 59.7 | (51.8-67.6) | 0.13 | (0.09-0.18) |
| 50-54 | 228 | 15.0 | (10.4-19.6) | 31.1 | (24.7-37.5) | 53.9 | (46.5-61.3) | 0.10 | (0.08-0.13) |
| 55-59 | 251 | 8.8 | (4.0-13.6) | 38.8 | (32.1-45.5) | 52.4 | (46.0-58.8) | 0.10 | (0.08-0.12) |
| 60-64 | 216 | 10.3 | (6.2-14.4) | 52.3 | (43.8-60.8) | 37.4 | (29.9-44.9) | 0.07 | (0.06-0.09) |
| 65-69 | 200 | 26.5 | (19.6-33.4) | 39.4 | (30.4-48.4) | 34.1 | (26.2-42.0) | 0.05 | (0.04-0.06) |
| 70-74 | 193 | 46.2 | (36.9-55.5) | 26.6 | (19.2-34.0) | 27.1 | (19.2-35.2) | 0.03 | (0.02-0.04) |
| 75-79 | 156 | 45.0 | (35.8-54.2) | 26.2 | (17.8-34.6) | 28.8 | (20.8-36.8) | 0.03 | (0.02-0.04) |
| **Women** | | | | | | | | | |
| <1 | 83 | 4.2 | (0.0-8.9) | 26.6 | (13.7-39.5) | 69.3 | (56.7-81.9) | 0.19 | (0.13-0.27) |
| 1-4 | 333 | 2.2 | (0.5-3.9) | 22.2 | (16.7-27.7) | 75.6 | (70.0-81.2) | 0.30 | (0.25-0.37) |
| 5-9 | 252 | 1.8 | (0.0-3.7) | 23.8 | (16.2-31.4) | 74.5 | (68.2-80.8) | 0.29 | (0.24-0.36) |
| 10-14 | 294 | 0.3 | (0.0-0.8) | 18.6 | (12.9-24.3) | 81.1 | (75.4-86.8) | 0.29 | (0.24-0.33) |
| 15-19 | 243 | 0.7 | (0.0-1.7) | 28.2 | (21.9-34.5) | 71.1 | (64.8-77.4) | 0.17 | (0.15-0.20) |
| 20-24 | 199 | 2.8 | (0.4-5.2) | 31.3 | (23.6-39.0) | 65.9 | (58.3-73.5) | 0.14 | (0.12-0.18) |
| 25-29 | 226 | 5.0 | (1.5-8.5) | 27.1 | (20.3-33.9) | 67.9 | (60.5-75.3) | 0.14 | (0.11-0.18) |
| 30-34 | 244 | 10.0 | (5.7-14.3) | 36.9 | (30.5-43.3) | 53.0 | (47.3-58.7) | 0.11 | (0.09-0.13) |
| 35-39 | 282 | 5.6 | (2.5-8.7) | 37.0 | (29.6-44.4) | 57.4 | (50.6-64.2) | 0.13 | (0.11-0.15) |
| 40-44 | 247 | 8.7 | (4.8-12.6) | 24.4 | (18.5-30.3) | 66.9 | (60.9-72.9) | 0.16 | (0.12-0.20) |
| 45-49 | 261 | 22.0 | (15.8-28.2) | 37.6 | (30.6-44.6) | 40.3 | (32.3-48.3) | 0.06 | (0.04-0.08) |
| 50-54 | 264 | 28.2 | (20.2-36.2) | 41.7 | (34.5-48.9) | 30.1 | (23.0-37.2) | 0.04 | (0.03-0.05) |
| 55-59 | 250 | 22.3 | (16.3-28.3) | 49.7 | (43.6-55.8) | 28.0 | (22.3-33.7) | 0.04 | (0.03-0.05) |
| 60-64 | 237 | 29.2 | (22.9-35.5) | 45.9 | (39.7-52.1) | 24.8 | (19.0-30.6) | 0.03 | (0.02-0.04) |
| 65-69 | 263 | 38.0 | (30.6-45.4) | 34.3 | (29.0-39.6) | 27.7 | (21.3-34.1) | 0.03 | (0.02-0.05) |
| 70-74 | 218 | 58.2 | (50.8-65.6) | 25.0 | (18.9-31.1) | 16.8 | (11.0-22.6) | 0.02 | (0.01-0.02) |
| 75-79 | 175 | 52.7 | (43.8-61.6) | 26.6 | (19.4-33.8) | 20.7 | (12.7-28.7) | 0.02 | (0.01-0.03) |

[a]CI, confidence interval.

percentage with full protection decreased after the age of 10 to 14 years, but increased for the 35- to 44-year age group (Figure 1). After the age of 40 to 44 years, the percentage with full protection and the geometric mean titer decreased. Although the geometric mean titers differed statistically significantly by gender only after the age of 30 years, they were slightly lower for females 5 to 9 years of age and older (Table 3).

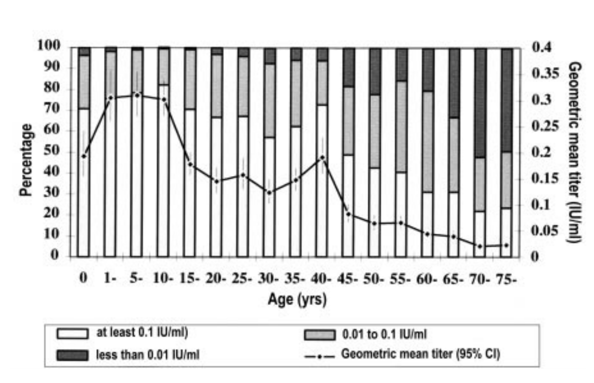

Figure 1. Age-specific diphtheria immunity in a nationwide sample (n = 7715), the Netherlands.

Both for orthodox reformed persons less than 50 years and for those at least 50 years of age, the proportion with no protection was higher than for persons in the nationwide sample (Table 4).

Men and women ages 20 to 49 years without a military service history had similar proportions of full, basic, and no protection, while the proportion with full protection was higher for men with a military service history (Table 5).

### Persistence of Diphtheria Antitoxin Levels

The geometric mean titer decreased with age (or time since last vaccination) for persons who had received their sixth and last vaccination at 8 to 9 years of age (n = 961) from 0.30 IU/ml for 10 to 14 years to 0.09 IU/ml for 30 to 34 years (Table 6, Figure 2). According to linear regression analysis, the decrease corresponds to a decrease of -1.27 $^{2}$log IU/ml with each $^{2}$log increase in years. The percentage with full protection decreased from 82.5% to 41.7%, and the percentage with no protection increased from 0% to 4.3% for persons 10 to 14 years of age and 30 to 34 years of age, respectively (Table 6).

The geometric mean titer for persons 20 to 34 years of age with documented revaccination (n = 37) was 0.29 IU/ml. Percentages of full (81.0%), basic (19.0%), and no protection (0.0%) were similar to recently vaccinated 10- to 14-year-olds, without further documented or reported revaccination (Table 6).

### Conclusions

Our population-based study showed that 58% of the Dutch population had full, 30% basic, and 12% no protection against diphtheria. These estimates and the geometric mean titer (0.12 IU/ml) are in between findings for other European countries (4,11-20). The Dutch immunization program appeared to induce long-term protection. However, approximately one third of adults age 50 to 79 years, who were born before the introduction of the immunization program, and approximately two-thirds of orthodox reformed persons had no protective diphtheria antibodies.

Table 4. Diphtheria immunity in a nationwide sample and in orthodox reformed persons in municipalities with low vaccine coverage, the Netherlands.

| Sample[a] (yrs) | No. | <0.01 IU/ml | (95% CI[b]) | 0.01-0.1 IU/ml | (95% CI) | ≥0.1 IU/ml | (95% CI) | Geometric mean titer | (95% CI) |
|---|---|---|---|---|---|---|---|---|---|
| **NW** | | | | | | | | | |
| 0-49 | 5,064 | 5.3 | (4.4-6.2) | 27.3 | (25.3-29.4) | 67.4 | (65.2-69.5) | 0.17 | (0.16-0.19) |
| 50 -79 | 2,651 | 29.2 | (26.4-31.9) | 37.4 | (35.3-39.5) | 33.4 | (31.3-35.6) | 0.04 | (0.04-0.05) |
| **ORLVC** | | | | | | | | | |
| 0-49 | 170 | 60.8 | (40.2-81.4) | 7.4 | (2.3-12.6) | 31.8 | (13.8-49.7) | 0.02 | (0.01-0.05) |
| 50 -79 | 63 | 59.3 | (30.1-88.6) | 34.6 | (9.5-59.6) | 6.1 | (0.0-18.2) | 0.01 | (0.005-0.025) |

[a]NW, nationwide; ORLVC, Orthodox reformed from low vaccine coverage sample.
[b]CI, confidence interval.

Table 5. Diphtheria immunity in a nationwide sample among persons 20 to 49 years of age, according to sex and military service, the Netherlands

| Sample[a] | No. | <0.01 IU/ml | (95% CI[b]) | 0.01-0.1 IU/ml | (95% CI) | ≥0.1 IU/ml | (95% CI) | Geometric mean titer | (95% CI) |
|---|---|---|---|---|---|---|---|---|---|
| M, SH | 425 | 2.9 | (1.2-4.6) | 14.9 | (11.1-18.7) | 82.2 | (78.0-86.5) | 0.30 | (0.25-0.36) |
| M, NSH | 710 | 7.8 | (5.2-10.3) | 33.1 | (28.0-38.1) | 59.2 | (54.0-64.4) | 0.12 | (0.10-0.14) |
| W, NSH | 1,456 | 9.1 | (7.0-11.2) | 32.5 | (29.0-36.0) | 58.4 | (54.5-62.3) | 0.12 | (0.10-0.13) |

[a]M, men; W, women; SH, service history; NSH, no service history.
[b]CI, confidence interval.

Table 6. Diphtheria immunity for persons in a nationwide sample who were completely vaccinated in the national immunization program and received the sixth diphtheria vaccination at 8 or 9 years of age, the Netherlands

| Sample[a] (yrs) | No. | <0.01 IU/ml | (95% CI[b]) | 0.01-0.1 IU/ml | (95% CI) | ≥0.1 IU/ml | (95% CI) | Geometric mean titer (IU/ml) | (95% CI) |
|---|---|---|---|---|---|---|---|---|---|
| **NRE** | | | | | | | | | |
| 10-14 | 392 | 0.0 | -- | 17.5 | (12.8-22.1) | 82.5 | (77.9-87.1) | 0.30 | (0.26-0.34) |
| 15-19 | 282 | 0.4 | (0.0-1.3) | 30.1 | (23.7-36.5) | 69.4 | (63.2-75.7) | 0.17 | (0.14-0.21) |
| 20-24 | 155 | 1.2 | (0.0-3.0) | 29.4 | (20.7-38.2) | 69.4 | (60.7-78.1) | 0.16 | (0.12-0.19) |
| 25-29 | 80 | 1.6 | (0.0-3.7) | 28.3 | (17.2-39.4) | 70.2 | (58.7-81.6) | 0.14 | (0.11-0.18) |
| 30-34 | 52 | 4.3 | (0.0-9.2) | 54.0 | (39.3-68.8) | 41.7 | (27.3-56.1) | 0.09 | (0.07-0.13) |
| **RE** | | | | | | | | | |
| 20-34 | 37 | 0.0 | -- | 19.0 | (3.2-34.9) | 81.0 | (65.1-96.8) | 0.29 | (0.18-0.46) |

[a]NRE, no evidence of revaccination; RE, evidence of revaccination.
[b]CI, confidence interval.

The toxin inhibition test used to measure diphtheria antitoxin concentrations shows good correlation with the in vitro neutralization test in Vero cells, but is faster, simpler, and combines the measurement of diphtheria and tetanus antitoxin antibodies (8,9).

Although the participation rates in the nationwide sample and low vaccine coverage sample were 55% and 52.5%, respectively, our population-based estimates of diphtheria immunity were considered representative, because they do not seem to be affected by nonparticipation. Our participants included a large percentage of persons with diphtheria protection who were born after the vaccination was introduced in 1952 and after the virtual disappearance of diphtheria in 1960. High levels of immunity in this group reflect the success of the national vaccination program.

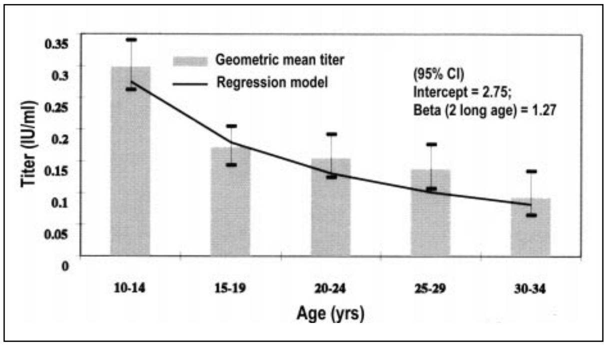

Figure 2. Diphtheria antitoxin titer (geometric mean titer ± 2 standard errors) by age group and linear regression of diphtheria antitoxin antibody titer (in [2]log) with age (in [2]log years) for persons who received the sixth diphtheria vaccination at the age of 8 or 9 years (n = 961) in the nationwide sample, the Netherlands.

For persons born before the introduction of vaccination, diphtheria immunity is largely derived from natural infection. However, immunity levels in persons older than 49 years in the general population are higher than those of orthodox reformed persons, suggesting that immunity was partly induced by vaccinations (e.g., for military service, travel).

The sharp increase in the percentage of persons older than 44 years with no protective diphtheria antitoxin levels is consistent with findings of other studies (4,11,12,14-19). The increase supports the phenomenon of waning immunity after natural infection without boosting.

In our study, higher immunity levels among men are associated with military service, as previously reported (15,19). However, some researchers have found similar immunity levels for men and women, while others have reported lower immunity for men (11,16,21). Furthermore, lower immunity for women that could not be ascribed to vaccinations during military service has also been reported (4,20). Women might maintain immunity after vaccination for a shorter time than men (14). The slightly lower geometric mean titers for girls age 5 to 19 years in our study are consistent with the latter possibility. As more than 60% of orthodox reformed persons have no protection against diphtheria, introduction of diphtheria into this group may constitute a danger of spreading the bacterium.

Since the Netherlands does not have a mandatory vaccination policy, protection of persons who refuse vaccination is problematic. For poliomyelitis the solution seems to be eradication of the causative agent (5). For diphtheria such a goal has not yet been formulated by WHO. However, even though systematic assessment has not been performed, no signs of persistent circulation of *C. diphtheriae* exist in the Netherlands.

When our data are interpreted longitudinally, the log linear decrease in diphtheria antibody level with age for completely vaccinated persons corresponds with a continuous decline in vaccine-induced antibodies (13,22). However, relatively few 30- to 34-year-old persons (4.3%) who received their last vaccination approximately 25 years ago had a diphtheria antitoxin level of less than 0.01 IU/ml. This compares favorably with observations in other countries (13,21-23). Our immunization program, in which children are vaccinated at 3, 4, 5, and 11 months with 15 Lf diphtheria toxoid and at the ages of 4 and 9 years with 2.5 Lf, appears to induce long-term protection against diphtheria.

In the Netherlands, booster vaccinations are only advised for persons at increased risk for exposure (e.g., travelers to endemic-disease countries and those who work with injection drug users and alcoholic patients). The need for routine boosters to guarantee population protection depends mainly on the proportion of vaccinated persons necessary to confer diphtheria herd immunity. This proportion is estimated at 70% to 80%, but no antitoxin level has been precisely defined for complete protection (10,13,24-26). The Dutch immunity level exceeds this threshold (a minimum level of 0.01 IU/ml [88%]), but is below a minimum level of 0.1 IU/ml (58%).

The absence of cases in the Netherlands associated with the diphtheria epidemic in Eastern Europe suggests that herd immunity is sufficient. This herd immunity might result from sufficient protective levels of antitoxin or immunologic memory. Our results, like those of others, indicate good immunologic memory after revaccination for persons who had been previously vaccinated (17, 27). However, the memory response of adults after initial vaccination is unknown. Furthermore, unknown protective mechanisms might be involved. Only sporadic cases and no outbreaks have occurred in other European countries where gaps have been found in the diphtheria antitoxin levels of adults. The only recent epidemic in western Europe, which occurred before the epidemic in eastern Europe, was among alcoholics (23). Perhaps unfavorable social conditions, like those that appear to have contributed to the epidemics in eastern Europe, play a role in the spread of diphtheria.

In conclusion, a substantial percentage of adults born before the introduction of the immunization program has low diphtheria antitoxin levels. Although herd immunity seems sufficient, long-term population protection cannot be assured. Possibly vaccination might fill the gaps of diphtheria antitoxin antibodies. Diphtheria vaccination could be efficiently combined with other vaccines (e.g., tetanus, influenza) as part of an adult immunization program.

### Acknowledgments

We thank the Public Health Services, the Pienter Project Team, and C.J.P. van Limpt and H.A.T. Kuijken for their useful contributions. We also thank J. Huisman, H.C. Rümke, J.F.P. Schellekens, and J.K. van Wijngaarden for their valuable comments on our manuscript.

H.E. de Melker is an epidemiologist in the Department of Infectious Diseases Epidemiology, National Institute of Public Health and the Environment, the Netherlands. Her work involves epidemiologic research directed to vaccine-preventable diseases and evaluation of the national vaccination program.

### References

1. Hardy IRB, Dittmann S, Sutter RW. Current situation and control strategies for resurgence of diphtheria in newly independent states of the former Soviet Union. Lancet 1996;347:1739-44.
2. World Health Organization. The expanded programme on immunisation in the European Region of WHO. Diphtheria. Plan of action for the prevention and control of diphtheria in the European Region (1994-1995). Copenhagen, 1994. ICP/EPI 038 (A).
3. Prospero E, Raffo M, Bagnoli M, Appignanesi R, D'Errico M. Diphtheria: epidemiological update and review of prevention and control strategies. Eur J Epidemiol 1997;13:527-34.
4. Maple PA, Efstratiou A, George RC, Andrews NJ, Sesardic D. Diphtheria immunity in UK blood donors. Lancet 1995;345:963-5.
5. Oostvogel PM, van Wijngaarden JK, van der Avoort HGAM, Mulders MN, Conyn-van Spaendonck MAE, Rümke HC, et al. Poliomyelitis outbreak in an unvaccinated community in the Netherlands, 1992-93. Lancet 1994;344:665-70.
6. de Melker HE, Conyn-van Spaendonck MAE. Immunosurveillance and the evaluation of national immunisation programmes: a population-based approach. Epidemiol Infect 1998;21:637-43.
7. Cochran WG. Sampling techniques. 3rd ed. New York: John Wiley & Sons; 1977.
8. Hendriksen CFM, van de Gun JW, Kreeftenberg JG. Combined estimation of tetanus and diphtheria antitoxin in human sera by the in vitro Toxin Binding Inhibition (ToBI) test. Journal of Biological Standardization 1989;17:191-200.
9. Knipping CT, Berbers GAM. Vergelijking van de Toxine Bindings Inhibitie test (ToBI) met de Dubbel Antigeen ELISA (DAE) voor het bepalen van antistoftiters gericht tegen difterie toxine in humaan serum. RIVM report no. 126000001, 1998, Bilthoven, the Netherlands.
10. Galazka AM. The immunological basis for immunisation. Diphtheria. Geneva: World Health Organization. Geneva; 1993. WHO/EPI/GEN/93.12.
11. Stark K, Barg J, Molz B, Vormwald A, Bienzle U. Immunity against diphtheria in blood donors in East Berlin and West Berlin. Lancet 1997;350:932.
12. Matheï C, van Damme P, Bruynseels P, Goossens H, Vranckx R, Metheus A. Diphtheria immunity in Flanders. Eur J Clin Microbiol Infect Dis 1997;16:631-6.
13. Simonsen O, Kjeldsen K, Bentzon MW, Heron I. Susceptibility to diphtheria in populations vaccinated before and after elimination of indigenous diphtheria in Denmark. A comparative study of antitoxic immunity. Acta Pathol Microbiol Immunol Scand Sect C 1987;95:225-31.
14. Kjeldsen K, Simonsen O, Heron I. Immunity against diphtheria and tetanus in the age group 30-70 years. Scand J Infect Dis 1988;20;177-85.
15. Jenum PA, Skogen V, Danilova E, Eskild A, Sjursen H. Immunity to diphtheria in northern Norway and northwestern Russia. Eur J Clin Microbiol Infect Dis 1995;14:794-8.
16. Klouche M, Lühmann D, Kirchner H. Low prevalence of diphtheria antitoxin in children and adults in northern Germany. Eur J Clin Microbiol Infect Dis 1995;14:682-5.
17. World Health Organization. Expanded programme on immunization. Immunization of adults against diphtheria. Wkly Epidemiol Rec 1995;70:56-9.
18. World Health Organization. Expanded programme on immunization. Diphtheria immunity in the adult French population. Wkly Epidemiol Rec 1995;70:252-5.
19. Gasparini R, Pozzi T, Fragapane E, Severini R, Cellesi C, Fabrizi P, et al. Immunity to diphtheria in Siena. Epidemiol Infect 1997;119:203-8.
20. Miller E, Rush M, Morgan-Capner P, Hutchinson D, Hindle L. Immunity to diphtheria in adults in England. BMJ 1994;308:598.
21. Cellesi C, Michelangeli C, Rossolini GM, Giovannoni F, Rossolini A. Immunity to diphtheria, six to 15 years after a basic three-dose immunization schedule. Journal of Biological Standardization 1989;17:29-34.
22. Kjeldsen K, Simonsen O, Heron I. Immunity against diphtheria 25-30 years after primary vaccination in childhood. Lancet 1985;1:900-2.
23. Böttiger M, Pettersson G. Vaccine immunity to diphtheria: a 20-year follow-up study. Scand J Infect Dis 1992;24:753-8.
24. Anderson RM, May RM. Infectious diseases of humans: dynamics and control. 2nd ed. New York: Oxford University Press; 1991.
25. Ipsen J. Circulating antitoxin at the onset of diphtheria in 425 patients. J Immunol 1946;54:325-47.
26. Schneerson R, Robbins JB, Taranger J, Lagergärd T, Trollfors B. A toxoid vaccine for pertussis as well as diphtheria? Lessons to be relearned. Lancet 1996;348:1289-92.
27. Simonsen O, Kjeldsen K, Vendborg HA, Heron I. Revaccination of adults against diphtheria I: response and reactions to different doses of diphtheria toxoid in 30-70-year-old persons with low serum antitoxin levels. Acta Pathol Microbiol Immunol Scand Sect C 1986;94:213-8.
